# Potentially traumatic events and the association with hazardous alcohol use in 19,128 middle aged and elderly adults: the Tromsø Study 2015–2016

**DOI:** 10.1007/s00127-024-02801-3

**Published:** 2024-12-18

**Authors:** Vendela Husberg-Bru, Laila A. Hopstock, Jens C. Thimm, Torgeir Gilje Lid, Kamilla Rognmo, Catharina Elisabeth Arfwedson Wang, Kristin Gustavson

**Affiliations:** 1 Department of Psychology, UiT The Arctic University of Norway, Langnes, P.O. Box 6050, 9037 Tromsø, Norway; 2 KORFOR - Center for Alcohol and Drug Research, Stavanger University Hospital, Stavanger, Norway; 3 Department of Public Health, Faculty of Health Sciences, University of Stavanger, Stavanger, Norway; 4 Department of Health and Care Sciences, UiT The Arctic University of Norway, Tromsø, Norway; 5 Centre for Crisis Psychology, University of Bergen, Bergen, Norway; 6 Department of Psychology, University of Oslo, Oslo, Norway; 7 Norwegian Institute of Public Health, Oslo, Norway

**Keywords:** Hazardous alcohol use, Potentially traumatic events, Population-based study, AUDIT

## Abstract

**Purpose:**

The aim was to examine the association between a wide range of potentially traumatic events (PTEs) experienced in childhood, adulthood or both, and hazardous alcohol use, including the relationship between the total sum of PTEs and hazardous alcohol use in middle aged and elderly adults***.*** Previous studies have predominantly focused on childhood PTEs or isolated PTEs and more severe alcohol problems, little focus has been given to middle aged and elderly adults with hazardous alcohol use and PTE experiences.

**Methods:**

We used logistic regression analysis to study the relation between a broad range of PTEs and hazardous alcohol defined by the alcohol use disorder identification test (AUDIT) in 19,128 women and men aged 40 years and above participating in the seventh survey of the Norwegian population-based Tromsø Study in 2015–2016. Alcohol abstainers were excluded from the analyses.

**Results:**

Experience of violence, sexual abuse, bullying, painful or frightening medical and dental treatments, and serious illness or accident by a loved one were associated with higher odds for hazardous alcohol use. Further, there were higher odds of hazardous alcohol use per additional experienced PTE (*OR* = 1.22, 95% CI 1.20–1.25, *p* ≤ 0.001).

**Conclusion:**

PTEs were prevalent among participants who had a hazardous alcohol use. Also, most of the PTEs occurring in childhood, adulthood or both were independently related to hazardous alcohol use. Moreover, the findings indicate an association in the relationship between the number of PTEs and hazardous alcohol use.

**Supplementary Information:**

The online version contains supplementary material available at 10.1007/s00127-024-02801-3.

## Introduction

Hazardous alcohol use refers to exceeding a threshold level of consumption associated with increased risk for adverse events and ill health [1]. On the spectrum of alcohol use disorders, hazardous alcohol use is on the less severe end, where prevention strategies towards reducing the alcohol intake may be effective [2]. Factors associated with hazardous alcohol use are being male [3], low socioeconomic status (SES), old age [4], and comorbidity with somatic and psychiatric disorders, such as insomnia [5], cardiovascular disease [6], some types of cancer [7], depression and anxiety [8], and drug use disorders [9]. Previous research has shown a relationship between potentially traumatic events (PTEs) and alcohol problems [10–12], meaning that individuals who have past PTE experiences have a higher probability of developing alcohol problems.

PTEs are experiences involving harm or threat [13, 14], such as sexual abuse, childhood neglect, violence, serious accidents, bereavement, or bullying, and are all associated with increased risk of high alcohol consumption [11] and alcohol problems [15–17]. Epidemiological studies have found a lifetime prevalence of PTEs ranging from 26.5% to 71.1% in adult samples [14, 15, 18, 19]. The World Mental Health Survey from 24 countries found a lifetime prevalence of PTEs at 70.4% [18]. This suggests that exposure to PTEs is common in the general population. The large variation in the prevalence of PTEs between studies may be due to differences in methodology, e.g., the number of PTEs that were assessed. Previous studies have primarily focused on single PTE categories, e.g., childhood trauma [20–22], interpersonal violence [12, 23], or bullying [24], samples of women [12] or men [25] only, or samples limited to college students [16] or clinical samples [26], neither of which are representative for the general population. Accordingly, it is necessary to examine the associations between a range of PTEs an hazardous alcohol use in the general population.

PTEs can be experienced in childhood and in adulthood [27]. PTEs experienced in adulthood may affect alcohol intake differently compared to PTEs experienced in childhood [28, 29]. Previous studies have found that experiencing PTEs in childhood may impact the psychosocial [30, 31] and physiological [32–34] development, and increase alcohol use later in life [35]. In general, childhood PTEs have received considerable attention [36–38], whereas studies of adulthood PTEs have primarily focused on specific PTEs, such as natural disasters [39, 40], terrorist attacks [29, 39], war/combat [41, 42], and physical and sexual abuse [43, 44]. Consequently, it is important to explore how different PTEs experienced in childhood or in adulthood may be differently associated with hazardous alcohol use.

Finally, previous studies have identified a relationship between the total sum of PTEs and later alcohol problems [10, 38]. Therefore, it is important to consider the possibility of a cumulative effect, i.e., whether having experienced several PTEs increases the odds of hazardous alcohol use.

More men than women have a hazardous alcohol use [45], and female gender is associated with having experienced more PTEs [14, 16, 46, 47]. Additionally, the proportion of elderly individuals drinking at hazardous levels are increasing [4]. It is thus relevant to adjust for sex and age when studying potential associations between PTEs and hazardous alcohol use.

The aims of this study were threefold: (i) examine the associations between a wide range of PTEs and hazardous alcohol use in women and men from a population-based sample, (ii) investigate whether there is a relationship between the total sum of PTEs experienced and hazardous alcohol use and (iii) investigate sex differences in (i) and (ii). We distinguished between PTEs experienced in childhood, or in adulthood and PTEs experienced both in childhood and in adulthood.

## Methods

### Study population

The Tromsø Study is a population-based study consisting of seven surveys (Tromsø1–Tromsø7 1974–2016) inviting inhabitants of the population of Tromsø municipality, Norway. Data collections include questionnaires and interviews, biological sampling, and clinical examinations [48, 49].

### Study sample

**Fig. 1:**
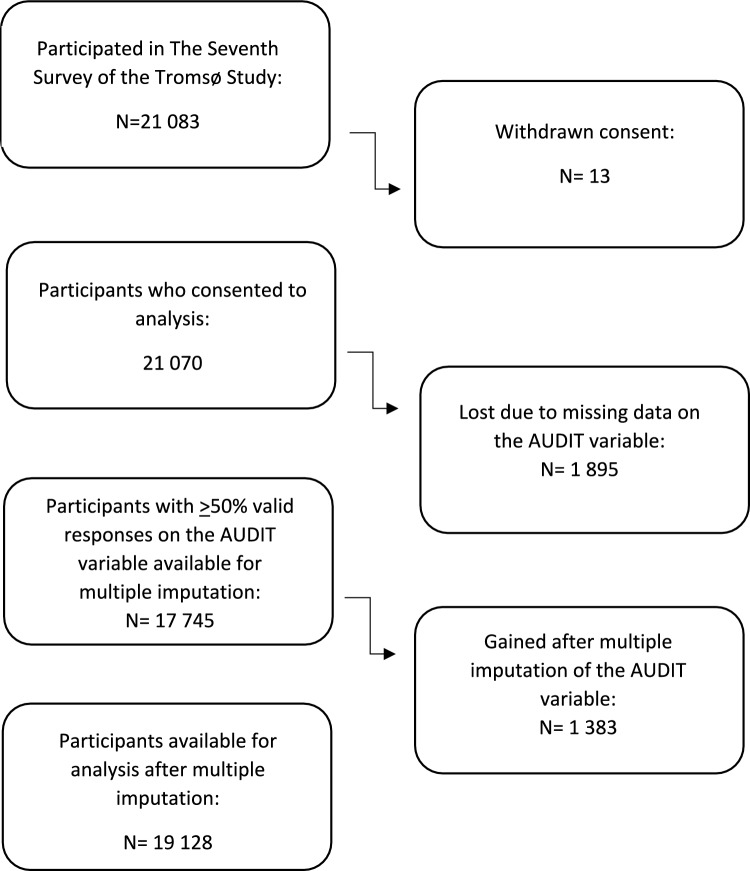
Flow chart illustrating the sample included in this study. The Tromsø Study (2015–2016)

The present study is based on questionnaire data from Tromsø7 (2015–2016) (53). Registered inhabitants in Tromsø municipality aged ≥ 40 years (N = 32,951) were invited, of which 65% attended (N = 21,070). Invitations were sent by mail and included an information brochure. To complete the online questionnaires, login credentials were provided [49]. Participants who reported no alcohol consumption during the 12 months prior to participating in the study were excluded. The sample included in the inferential analyses (results shown in Tables 5 and 6), after multiple imputation (MI, procedure described below) was N = 19,128. See Fig. 1 for a visual representation of the sample included in the inferential analyses (Fig. 1). As MI may cause ambiguous point estimates in descriptive statistics, all descriptive statistics shown are based on original data, which leads to different sample size in Tables 1, 2, 3, and 4 showing descriptive statistics (N between 17,745 and 21,070).

**Table 1:** Sample characteristics, stratified by sex

	Women (n = 11,064)	Men (n = 10,006)	p^a^
Mean age	57.3 (11.4)	57.4 (11.4)	0.220
Hazardous alcohol use	5.8 (522)	18.8 (1637)	< 0.001
Experienced childhood neglect	8.2 (887)	5.4 (501)	< 0.001

Potentially traumatic events	Childhood % (n)	Adulthood % (n)	Both childhood and adulthood % (n)	Childhood % (n)	Adulthood % (n)	Both childhood and adulthood % (n)	p
Serious illness/accident	4.1 (445)	16.0 (1722)	0.6 (68)	4.9 (478)	21.6 (2103)	1.1 (104)	< 0.001
Sexual abuse	(1096)	4.1 (438)	1.0 (108)	3.0 (292)	0.5 (46)	0.1 (< 5)	< 0.001
Violence	3.9 (418)	8.7 (931)	0.6 (66)	6.0 (587)	10.8 (1057)	1.5 (142)	< 0.001
Bullying	14.0 (1498)	6.7 (720)	1.5 (160)	15.5 (1511)	3.8 (375)	1.5 (144)	< 0.001
Witnessed violence	4.4 (472)	5.4 (575)	0.5 (52)	3.2 (314)	4.6 (445)	0.6 (63)	< 0.001
Other frightening, dangerous or violent events	3.2 (342)	4.1 (444)	0.2 (17)	2.4 (232)	6.7 (657)	0.2 (21)	< 0.001
Severe grief after bereavement	4.1 (438)	32.5 (3483)	1.7 (177)	3.5 (341)	23.3 (2272)	1.1 (108)	< 0.001
Painful medical treatment	3.4 (364)	8.0 (853)	0.3 (31)	3.1 (298)	6.9 (668)	0.2 (20)	0.004
Painful dental treatment	18.0 (1915)	5.3 (570)	1.0 (109)	17.5 (1706)	5.6 (548)	1.0 (102)	0.724
Serious illness/accident of a loved one	2.7 (289)	36.5 (3882)	1.7 (185)	2.6 (254)	28.1 (2722)	1.6 (161)	< 0.001

The Tromsø Study (2015–2016), N = 21,070—17,745^a^

N varies due to variability in missing data for the various variables. All descriptive statistics are based on unimputed data

Numbers are means (standard deviations) and proportions for continuous and categorical variables

Hazardous alcohol use = AUDIT score ≥ 8

Childhood neglect = yes/no

Childhood = before age 18

Adulthood = after age 18

^a^ significance test for age was F-test, for all other relationships Pearson Chi-square test was used

**Table 2:** The prevalence of potentially traumatic events in women, stratified by hazardous alcohol use

	No hazardous alcohol use, % (n)			Hazardous alcohol use, % (n)			p^a^
	Childhood	Adulthood	Experienced both in childhood and in adulthood	Childhood	Adulthood	Experienced both in childhood and in adulthood	
Serious illness or accident	4.2 (353)	15.8 (1339)	0.6 (50)	5.8 (30)	13.7 (71)	0.8 (4)	0.198
Sexual abuse	10.0 (844)	3.8 (320)	0.8 (68)	17.9 (92)	10.3 (53)	3.5 (18)	< 0.001
Violence	3.9 (327)	8.1 (685)	0.5 (41)	6.2 (32)	20.0 (104)	0.8 (4)	< 0.001
Bullying	13.7 (1163)	6.6 (557)	1.4 (119)	19.4 (100)	11.8 (61)	3.3 (17)	< 0.001
Witnessed violence	4.3 (366)	5.2 (441)	0.4 (35)	6.5 (34)	8.3 (43)	1.3 (7)	< 0.001
Other frightening, dangerous or violent events	2.6 (222)	3.8 (320)	0.1 (11)	1.4 (7)	6.2 (32)	0.2 (1)	0.015
Severe grief following bereavement	4.1 (348)	31.4 (2658)	1.6 (133)	5.0 (26)	33.7 (174)	2.1 (11)	0.273
Painful medical treatment	3.4 (289)	7.7 (655)	0.2 (20)	4.2 (22)	12.1 (63)	1.3 (7)	< 0.001
Painful dental treatment	18.1 (1529)	5.0 (429)	1.0 (87)	23.3 (120)	8.2 (42)	1.9 (10)	< 0.001
Serious illness or accident of a loved one	2.8 (235)	36.7 (3098)	1.7 (145)	3.5 (18)	39.3 (203)	2.7 (14)	0.112
Childhood neglect	No		Yes	No		Yes	
	92.4 (7871)		7.6 (644)	85.9 (445)		14.1 (73)	< 0.001

The Tromsø Study (2015–2016), n = 9059

Numbers are percentages for categorical variables

PTEs = potentially traumatic events

Hazardous alcohol use = alcohol use disorder identification test, score of ≥ 8. 6

Childhood = before age 18

Adulthood = after age 18

^a^ Significance test is Pearson Chi-square test

**Table 3:** The prevalence of potentially traumatic events in men, stratified by hazardous alcohol use

	No Hazardous alcohol use %, (n)			Hazardous alcohol use %, (n)			p^a^
	Childhood	Adulthood	Experienced both in childhood and in adulthood	Childhood	Adulthood	Experienced both in childhood and in adulthood	
Serious illness or accident	4.4 (310)	20.6 (1443)	0.8 (59)	6.7 (109)	23.8 (388)	2.2 (36)	< 0.001
Violence	5.5 (387)	9.0 (633)	0.9 (66)	8.7 (142)	19.4 (316)	4.1 (67)	< 0.001
Sexual abuse	2.2 (158)	0.3 (23)	0.0 (0)	5.6 (92)	1.0 (17)	0.2 (< 5)	< 0.001
Bullying	14.7 (1032)	3.3 (231)	1.2 (86)	19.5 (318)	5.9 (96)	2.6 (42)	< 0.001
Witnessed violence	2.5 (178)	4.2 (293)	0.5 (33)	6.1 (99)	6.7 (109)	1.5 (24)	< 0.001
Another frightening, dangerous or violent event	2.2 (154)	6.1 (428)	0.1 (9)	1.7 (28)	9.7 (158)	0.5 (8)	< 0.001
Severe grief following bereavement	3.3 (231)	22.1 (1554)	0.8 (59)	4.1 (67)	25.4 (415)	2.5 (40)	< 0.001
Painful medical treatment	2.5 (172)	6.3 (439)	0.1 (10)	5.3 (87)	8.1 (132)	0.6 (9)	< 0.001
Painful dental treatment	16.5 (1159)	5.1 (357)	0.9 (60)	22.9 (372)	7.7 (125)	1.8 (29)	< 0.001
Serious illness or accident of a loved one	2.5 (172)	26.9 (1886)	1.5 (102)	3.9 (64)	33.4 (543)	2.6 (43)	< 0.001
	No		Yes	No		Yes	
Childhood neglect	95.3 (6702)		4.7 (333)	92.0 (1503)		8.0 (131)	< 0.001

The Tromsø Study (2015–2016), n = 8 686.

Hazardous alcohol use = alcohol use disorder identification test (AUDIT) score of ≥ 8

Childhood neglect = Yes/No

^a^ Significance test is Pearson Chi-square test

**Table 4:** The total number of experienced potentially traumatic events among women and men

Number of PTEs	Women *n* = 9903		Men *n* = 9272		p^a^
	%	(n)	%	(n)	
0	21.1	(2094)	25.7	(2385)	< 0.001
1–2	44.4	(4397)	44.3	(4108)	
3–4	23.5	(2323)	21.9	(2033)	
5–7	9.7	(961)	7.0	(653)	
8 or more	1.3	(128)	1.0	(93)	

The Tromsø Study (2015–2016), n = 19,175

Numbers are percentages (n) for categorical variables

PTEs = Having experienced a severe illness or accident, violence, sexual abuse, bullying, witnessed violence or sexual abuse, another frightening, dangerous or violent event, childhood neglect, painful medical or dental treatment, and or serious illness/accident or severe grief following bereavement

^a^ Significance test is Pearson Chi-square test

### Measures

#### Hazardous alcohol use

The outcome variable, hazardous alcohol use, was defined as a pattern of alcohol intake that increases the risk of harmful consequences, measured with the Alcohol Use Disorder Identification Test (AUDIT). The AUDIT is a well-validated tool to measure hazardous and harmful alcohol use in the past 12 months [50]. It consists of ten items, with response categories ranging from 0 to 4 (sum score = 0–40) [2]. We used the AUDIT as a dichotomous variable with a cut-off score ≥ 8 which is indicative of hazardous alcohol use [51]. Few respondents scored in the higher regions of the AUDIT, thus hindering a fine-grained categorization. The binary variable includes participants with severe alcohol problems. However, the term hazardous alcohol use will be used in this paper. Multiple imputation was used to estimate missing values on the AUDIT items. The procedure is described below.

#### Potentially traumatic events (PTEs)

Respondents reported if they had experienced any of the following PTEs: (a) life threatening illness or serious accident, (b) violence, (c) sexual abuse, (d) bullying, (e) witnessed a loved one being exposed to violence or sexual abuse, (f) other frightening dangerous or violent events, (g) severe grief following bereavement, (h) painful or frightening medical treatment, (i) painful or frightening dental treatment, (j) childhood neglect (physical and emotional) and (k) serious illness/accident of a loved one. For a more detailed description of the PTE variables, see Thimm and colleagues [14].

Childhood neglect was a binary variable (yes/no). The other PTEs differentiated between experienced before the age of 18, after age 18 and/or in the last year. Except from childhood neglect, the PTEs were coded in the following way: experienced in childhood (before age 18), in adulthood (after age 18), and experienced both in childhood and in adulthood (experienced before and after age 18). This enabled investigations of the association between each PTE and hazardous alcohol use at one point during their life, from childhood to adulthood. Thus, PTEs experienced in childhood, adulthood and both in childhood and adulthood were the response categories as presented in this study. For simplicity, when discussing the results from each PTE we will refer to the three categories: (1) exposure in childhood, (2) exposure in adulthood, and (3) exposure both in childhood and in adulthood.

To investigate if there is a relationship between the number of experienced PTEs and hazardous alcohol use, all PTEs were summed into a variable scoring between 0 and 11, which was subsequently categorized into five categories; (0) 0 PTEs, (1) 1–2 PTEs, (2) 3–4 PTEs, (3) 5–7 PTEs and (4) 8 or more PTEs. This variable is from now on referred to as the number of PTEs.

#### Covariates

Age (continuous) and sex were included as covariates. To assess the relationship between each individual PTE and hazardous alcohol use, all PTEs were entered together into the fully adjusted model, serving as covariates for each other.

### Statistical analyses

Descriptive analyses were run on data from the full unimputed sample, stratified by sex and hazardous alcohol use. Chi-square test were used for all categorical variables in the descriptive statistics, and one-way ANOVA was run testing if age differed between women and men. N in the descriptive tables varies between 21,070 and 17,745, due to variability in missing values. Descriptive statistics are shown in Tables 1, 2, 3, and 4. We conducted a series of logistic regression analyses, with each PTE independently serving as a predictor variable. Then, the PTEs were entered into the same model to mutually adjust for each other’s association with hazardous alcohol use. We ran the following multiple logistic regression models: model 1a included one PTE as a predictor. Model 1b included the predictor as well as age and sex as covariates, as well as the interaction term between the PTE variable and sex. In model 1c, all PTEs were entered and adjusted for each other as well as for age and sex. The interaction term between PTE and sex was not included in model 1c. Results of the logistic regression analyses of each individual PTEs relationship to hazardous alcohol use are shown in Table 5.

**Table 5:** The association between potentially traumatic events and hazardous alcohol use

	OR	99% CI	p	OR	99% CI	p	OR	99% CI	p
Childhood neglect	1.51	1.26–1.89	< 0.001	1.68	1.35–2.08	< 0.001	1.05	0.83–1.34	5.75
Frightening, dangerous or violent event									
Before 18	0.66	0.42–1.04	0.020	1.03	0.64–1.66	0.859	0.71	0.43–1.19	0.808
After 18	1.93	1.55–2.39	< 0.001	1.58	1.27–1.98	< 0.001	1.14	0.89–1.45	0.176
Both	3.12	1.13–8.60	0.004	2.54	0.86–7.45	0.026	1.17	0.36–3.87	0.726
Illness or accidents									
Before 18	1.69	1.32–2.16	< 0.001	1.47	1.14–1.90	< 0.001	1.16	0.88–1.52	0.171
After 18	1.31	1.13–1.51	< 0.001	1.27	1.09–1.47	< 0.001	1.01	0.85–1.18	0.978
Both	2.98	1.86–4.79	< 0.001	2.33	1.42–3.82	< 0.001	1.08	0.61–1.90	0.739
Violence									
Before 18	2.22	1.77–2.78	< 0.001	1.70	1.34–2.15	< 0.001	1.35	1.04–1.33	0.002
After 18	2.85	2.43–3.34	< 0.001	2.42	2.05–2.85	< 0.001	1.91	1.60–2.30	< 0.001
Both	6.22	4.21–9.19	< 0.001	3.88	2.58–5.84	< 0.001	2.62	1.65–4.16	< 0.001
Sexual abuse									
Before 18	1.36	1.09–1.68	< 0.001	2.20	1.75–2.77	< 0.001	1.62	1.26–2.09	< 0.001
After 18	1.52	1.08–2.13	0.002	3.15	2.19–4.54	< 0.001	1.97	1.33–2.92	< 0.001
Both	2.39	1.29–4.23	< 0.001	5.28	2.78–10.02	< 0.001	2.82	1.38–5.73	< 0.001
Bullying									
Before 18	1.55	1.33–1.81	< 0.001	1.33	1.14–1.56	< 0.001	1.04	0.88–1.24	0.502
After 18	1.60	1.27–2.03	< 0.001	1.88	1.47–2.40	< 0.001	1.36	1.05–1.77	0.002
Both	2.41	1.65–3.52	< 0.001	2.05	1.38–3.05	< 0.001	1.09	0.70–1.71	0.600
Witnessed violence									
Before 18	1.88	1.46–2.42	< 0.001	1.88	1.44–2.45	< 0.001	1.24	0.92–1.67	0.060
After 18	1.61	1.27–2.04	< 0.001	1.58	1.24–2.02	< 0.001	1.06	0.81–1.38	0.602
Both	3.70	2.15–6.37	< 0.001	2.91	1.64–5.17	< 0.001	1.06	0.50–2.00	0.996
Severe grief after bereavement									
Before 18	1.18	0.88–1.58	0.141	1.36	1.01–1.85	.009	1.12	0.81–1.53	0.350
After 18	1.01	0.88–1.15	0.877	1.29	1.12–1.48	< 0.001	1.10	0.95–1.28	0.100
Both	1.94	1.29–2.91	< 0.001	2.30	1.49–3.53	< 0.001	1.57	0.97–2.54	0.020
Painful medical treatment									
Before 18	1.79	1.36–2.37	< 0.001	1.89	1.41–2.52	< 0.001	1.33	0.97–1.83	0.020
After 18	1.37	1.11–1.68	< 0.001	1.49	1.20–1.84	< 0.001	1.12	0.88–1.42	0.200
Both	4.14	1.92–8.97	< 0.001	4.71	2.07–10.74	< 0.001	2.81	1.11–7.12	0.004
Painful dental treatment									
Before 18	1.49	1.29–1.73	< 0.001	1.54	1.33–1.79	< 0.001	1.30	1.11–1.52	< 0.001
After 18	1.71	1.36–2.15	< 0.001	1.64	1.30–2.08	< 0.001	1.36	1.6–1.75	0.002
Both	2.25	1.42–3.58	< 0.001	2.06	1.27–3.35	< 0.001	1.39	0.82–2.37	0.109
Illness by close one									
Before 18	1.56	1.14–2.15	< 0.001	1.53	1.10–2.14	< 0.001	1.24	0.87–1.76	0.115
After 18	1.18	1.04–1.34	< 0.001	1.33	1.17–1.52	< 0.001	1.11	0.97–1.28	0.049
Both	1.82	1.24–2.66	< 0.001	1.70	1.14–2.53	< 0.001	0.93	0.59–1.46	0.694

N = 19,128. The Tromsø Study (2015–2016)

The estimates are pooled results from multiple imputed datasets

Hazardous alcohol use = alcohol use disorder identification test (AUDIT) ≥ 8

Childhood neglect = yes/no

1^b^ Adjusted for age and sex, and the interaction between each individual PTE and sex. As the interaction terms, except one, were non-significant, these are not displayed in the tables

1^c^ Adjusted for age and sex, and all PTEs included in the analysis

^*^ α level = 0.01

*OR* Odds ratio, *CI* confidence interval

We also used multiple logistic regression to examine a potential relationship between the number of PTEs and hazardous alcohol use. Model 2a included the total sum of all PTEs as the predictor variable. Model 2b included the number of PTEs adjusted for age and sex. A third model (model 2c) was tested, including sex, age and the interaction term between the number of PTEs and sex. The results of the logistic regression analysis of the number of PTEs as a predictor of hazardous alcohol use is shown in Table 6.

**Table 6:** The association between the number of experienced potentially traumatic events and hazardous alcohol use

	Model 2a			Model 2b^a^		
Number of PTEs	OR	95% CI	p	OR	95% CI	p
1–2	1.26	1.11–1.43	< 0.001	1.32	1.16–1.51	0.028
3–4	1.99	1.73–2.28	< 0.001	2.13	1.85–2.45	< 0.001
5–7	2.78	2.36–3.28	< 0.001	3.15	2.65–3.74	< 0.001
8 or more	4.72	3.46–6.45	< 0.001	5.24	3.76–7.30	< 0.001
Sex				4.36	3.93–4.84	< 0.001
Age				0.96	0.96–0.97	< 0.001

The Tromsø study (2015–2016), n = 19,128

The estimates are pooled results from a multiple imputed dataset

Model 2a = included the number PTEs variable

Model 2b = included the PTEs variable, sex and age

^a^ The results of model 2c, including the interaction term are not shown, as the coefficients were only marginally altered and the interaction was non-significant

*OR* Odds ratio

Multicollinearity between the PTE variables was checked. All tolerance values were above 0.10 and the variance inflation factor (VIF) was less than 10 for all the PTE predictors, not indicating multicollinearity. To reduce the probability for type 1 error after testing multiple associations between different PTEs and hazardous alcohol use, the alpha level was increased to *p* ≤ 0.01, for the 11 logistic regression analyses with each PTE predictor variable. The alpha level was *p* ≤ 0.05 for the analysis concerning the total sum of PTEs and hazardous alcohol use, as this did not involve multiple tests. For the regression analyses, we included participants with valid data on the variables of interest after multiple imputation, which resulted in different sample sizes across the regression analysis due to listwise deletion (N ranging from 18,915 to 19,119). Effect sizes are presented as odds ratios (OR) with 99% and 95% confidence intervals (CI). All analyses were performed in SPSS version 28.

### Treatment of missing values

Hazardous alcohol use had a high proportion of missing values (8.0%), posing a risk of bias. We handled this with imputing missing data on respondents with partial data. Only respondents with information on at least ≥ 50% of the AUDIT items were included in the imputations. Respondents with > 50% missing data on the AUDIT were excluded from the regression analyses. Participants who abstained from alcohol in the past year (n = 1895, 8.1%) did not receive five of the items from the AUDIT in the online questionnaire. Accordingly, they had > 50% naturally missing items and were thus not included in the analysis. Multiple imputation is generally recommended over listwise deletion, and found to reduce the chance of bias [52, 53]. The imputations were performed with multiple imputation (fully conditional specification), with 20 imputed datasets. Age, sex, and the PTE variables, education, and symptoms of mental distress (Hopkins Symptoms Checklist-10) [54], were used as predictors in the imputations. The latter two variables were auxiliary variables not included in the regression analyses but used to estimate missing values. This resulted in a dataset including 19,128 participants, compared to 17,745 prior to multiple imputation. The results from the regression analyses in this study are presented from multiple imputed datasets. However, the same regression modelling was also performed on the non-imputed original dataset (n = 21,070).

## Results

### Descriptive statistics

Participants in the full sample (n = 21,070) had a mean age of 57 years (range 40–99 years), and 52% were women. The prevalence of hazardous alcohol use was 12.2%, 5.8% and 18.8% in women and men, respectively (see Table 1). Painful or frightening dental treatment, bullying, and sexual abuse were the most frequently reported childhood PTEs in women. In men, the most frequent childhood PTEs were painful or frightening dental treatment, bullying, and violence. Serious illness or accident of a loved one and severe grief following bereavement were the most common PTEs experienced in adulthood in both women and men. The PTE with the largest sex difference in prevalence was sexual abuse, with more than three times as many women having experienced sexual abuse in childhood compared to men. Women also experience bullying in adulthood almost twice as frequently as men. The differences in prevalence were significantly different between men and women for all PTEs, except painful dental treatment. Further sample characteristics are presented in Table 1 and sample characteristics for the imputed dataset (n = 19,128) is shown in supplementary material (Supplementary Table 1). When stratified on hazardous alcohol use (yes/no), serious illness or accident of a loved one and severe grief following bereavement remained the most prevalent PTEs, but the prevalences were not significantly different between women with and without hazardous alcohol use. Participants above cut-off for hazardous alcohol use had a higher prevalence of reported sexual abuse, violence, and bullying (Tables 2 and 3). The prevalence of sexual abuse was about twice as high in childhood for women and men with hazardous alcohol use, compared to women and men without hazardous alcohol use, whereas the prevalence of sexual abuse in adulthood was almost three times as prevalent for women and men with hazardous alcohol use, compared to the reference group. Violence in adulthood was about twice as prevalent in women and men with hazardous alcohol use, compared to women and men without. Overall, 75.1% of the respondents reported at least one PTE (Table 4).

### Logistic regression of each individual PTE and hazardous alcohol use

Logistic regression analyses on the multiple imputed dataset (n = 19,128) showed that after adjustment for sex and age (model 1b), the following PTEs were significantly associated with hazardous alcohol use in childhood, adulthood and both childhood and adulthood: childhood neglect (only measured in childhood), illness or accidents, violence, sexual abuse, bullying, witnessed violence, death of someone close painful medical treatments, painful or frightening dental treatment. Having experienced something other frightening, dangerous or violent event was significantly related to hazardous alcohol use if occurring in adulthood or both in childhood and adulthood (Table 5).

When exploring each PTEs individual contribution to the relationship to hazardous alcohol use, by adding all PTEs as covariates in model 1c, the results were somewhat altered (see Table 5). Of PTEs experienced in adulthood, violence, sexual abuse, bullying, painful dental treatment and illness of someone close were still significantly related to hazardous alcohol use. Of PTEs experienced in childhood, violence, sexual abuse, painful medical or dental treatment were significant. Having experienced violence, sexual abuse, severe grief after bereavement and painful medical treatments in both childhood and adulthood were also significantly related to hazardous alcohol use (Table 5). The odds ratios of having experienced violence, sexual abuse and painful medical treatments in both adulthood and childhood were considerably higher compared to the odds ratios of having the same experience in either childhood or adulthood.

### Interaction effects

Interaction effects were explored for each individual PTE and sex, except for sexual abuse and sex since few men (n =  < 5) reported sexual abuse both in childhood and in adulthood. Only one interaction effect was significant, between sex and having experienced a life-threatening illness or a serious accident in adulthood after adjustment for age and sex (OR = 1.49, 99% CI 1.02–2.17,* p* = 0.007), and after adjusting for the other PTEs (OR = 1.62, 99% CI 1.09–2.41, *p* = 0.002) (results not displayed in tables). The interaction indicated that the relationship between having experienced a life-threatening illness or accident in adulthood and hazardous alcohol use, was stronger among men than among women. There were no other significant interactions between PTEs and sex.

The number of PTEs were significantly related to hazardous alcohol use. The results presented in Table 6 clearly indicate increased odds of hazardous alcohol use with an increase in the number of PTEs, after adjustment for age and sex (see Table 6). The increase in odds approximated an additive effect—for each additional 2–3 PTE, the odds of hazardous alcohol use doubled or nearly doubled. The interaction between the number of PTEs and sex was not significant.

### Sensitivity analyses using original data

Sensitivity analyses of the non-imputed original dataset based on complete cases only (n = 21,070) were performed and showed mainly similar results as the results of the imputed data. After adjustment for sex, age and all other PTEs, violence (adulthood and both in childhood and adulthood) and sexual abuse (all three categories), bullying (in adulthood), painful or frightening medical (both in childhood and in adulthood) and dental treatments (in childhood, and in adulthood) were significantly related to hazardous alcohol use, much the same as in the pooled multiple imputed datasets. In addition, painful medical treatment in childhood was related to hazardous alcohol use in the non-imputed data (see table 2 in the Supplementary material).

## Discussion

The main findings from this population based study was that the odds for hazardous alcohol use was higher in participants reporting past experiences of violence, sexual abuse, painful medical or dental treatment, severe grief after bereavement or bullying in either childhood, adulthood or a combination of childhood or adulthood exposure. There was also an association between the number of PTEs and hazardous alcohol use, indicating that the odds of hazardous alcohol use about doubled with every 2–3 additional PTE experienced.

Participants who had experienced violence at any point during a lifetime, had higher odds for hazardous alcohol use. This is in line with previous findings [12, 55]. Results from the Adverse Childhood Experiences (ACE) study, which is based on health insurance records, showed that having experienced domestic physical abuse increased the risk for alcohol use disorders in adulthood [35]. A study using population-based data from Canada also found domestic violence to increase the odds for alcohol dependence [55]. Supplementing this, a population-based study of women [12] identified severe and moderate drinking to be highly associated with interpersonal violence (IPV). Most previous studies investigating violence and alcohol use primarily studied IPV [12, 23, 56–58]. Although IPV was not addressed in the current study, our findings supplement the previous findings suggesting that having experienced violence increased the odds of hazardous alcohol use.

Our results showed that participants who had experienced sexual abuse at any time (childhood, adulthood, or both), had higher odds of hazardous alcohol use. This corroborates the findings of previous studies [20, 59–61], although previous studies have primarily focused on sexual abuse in the context of adverse childhood experiences [20, 59–61]. However, some studies have studied sexual abuse experienced in adulthood. A study of women who had experienced violent assault (including sexual assault) found that alcohol levels increased following the assault [62]. These findings have been demonstrated in epidemiological [15, 61, 63, 64] and clinical studies [22, 26, 65]. For instance, patients with experiences of sexual abuse may report worse clinical symptoms of posttraumatic stress disorder and alcohol addiction, which can impact their treatment trajectories [26, 66, 67]. A recent clinical study revealed that women suffering from substance use disorders (SUD) (including problematic alcohol use) had a higher proportion of childhood traumas, compared to women in recovery from their SUD [65]. Additionally, one study using a large epidemiological dataset found that repeated experiences of sexual abuse was more common than experiencing it just once [43]. However, previous studies have largely focused on samples not representative of the general middle aged population, e.g. university students [67], or on elderly women-only samples [43].

The odds ratios for hazardous alcohol use were higher for individuals having experienced violence or sexual abuse in adulthood or both in childhood and adulthood, compared to only in childhood. As previous studies on the relationship between violence or sexual abuse and hazardous alcohol use most often have studied exposure to particular violent events occurring during either childhood (e.g. ACE) or adulthood (e.g. IPV), it is difficult to interpret this finding in light of previous research. Future studies should explore if exposure to violence or sexual abuse in adulthood has a stronger relationship to hazardous alcohol use than exposure to violence or sexual abuse occurring in childhood. It is, however, a noticeable stronger relationship between exposure to violence and sexual abuse in both childhood and adulthood compared to either exposure in childhood or adulthood. Although the confidence intervals in the current study overlapped in all but one case (violence in childhood vs violence in adulthood or both), one may expect multiple exposure to PTEs to be related to worse outcomes. This speculation is corroborated by the results of the logistic regression analyses presented in Table 6, indicating an increase in odds of hazardous alcohol use with increasing exposure to PTEs.

We found that experiences of bullying in adulthood yielded higher odds for hazardous alcohol use. One may speculate if adults who experience bullying use alcohol to cope. It may, also suggest that the association goes the other way, that adults who drink at hazardous levels experience more bullying, than adults who do not drink at hazardous levels. This is in line with a previous Norwegian study of workers, using registry data, who found that workplace bullying was associated with increased problematic alcohol use [68]. The majority of existing studies have investigated the relationship in the context of bullying experienced in early life and the development of alcohol problems in adolescence [69] or in adulthood [70, 71]. Future research should investigate the relationship between being bullied in adulthood and hazardous alcohol use further.

We also found that painful or frightening medical treatments both in childhood and in adulthood, and painful or frightening dental treatments in childhood was associated with hazardous alcohol use. Few previous studies have studied painful or frightening medical or dental treatment in relation to alcohol use, and it is thus difficult to make direct comparisons to our findings. It is important to acknowledge that the variable painful medical treatment is somewhat complex, as it may include subjective painful experiences from the treatment, but the treatment may also be an expression for an underlying disease or harm that in turn may increase hazardous alcohol use. To further complicate the matter, hazardous alcohol use may increase the likelihood of receiving medical treatment, painful or not, through enhancing the risk of various diseases [72]. This results is in line with a previous study of late middle-aged community residents, which found that participants with several painful medical conditions, consumed alcohol at more hazardous levels than participants without painful conditions [73]. The same was observed in a study of elderly individuals [74]. Also, one study identified an association between adult PTEs and persistent somatic symptoms [75], which may be related to higher odds of needing possibly painful medical procedures. Also, hazardous alcohol consumption over time increases the risk for developing somatic/mental diseases [76], which also may require painful medical treatments. We may speculate that this may partly explain our findings. Although there is a lack of existing research enabling a thorough understanding of the finding and acknowledging that there may be bidirectional effects as well as confounding factors (underlying somatic illness or injury) explaining the results, we believe this to be an important finding, providing knowledge regarding potentially detrimental effects of painful medical treatments, that may further enhance somatic and mental health problems. This finding needs to be corroborated by future studies.

In addition to the specific PTE predictors being associated with increased hazardous alcohol use, we also found a strong association between the number of PTEs and hazardous alcohol use. This is in line with previous studies [28, 38, 77]. For instance, several studies using data from the ACE study found a relationship between the number of ACEs experienced and alcohol problems in adulthood [28, 38, 77]. One study identified a cumulative relationship between experiencing childhood trauma and the development of alcohol abuse in adults and linked this to alcohol as one of the leading risks of mortality in adults [38]. Another finding from the ACE-study investigated the risk for liver disease in adults who had experienced childhood trauma and found that alcohol mediated a dose–response relationship between the traumatic experiences and liver disease [78].

In this study, childhood neglect, frightening or violent experiences, serious illness, or accidents, witnessing violence and illness by a close one, were associated with hazardous alcohol use. However, after adjusting for all PTEs in the final model 1c, these were no longer significant. The association between the number of experienced PTEs and hazardous alcohol use indicates a cumulative association. This may suggest that the PTEs deemed insignificant in model 1c might still influence the likelihood of hazardous alcohol use by contributing to the total number of PTEs encountered. The size of the odds ratios were small to moderate (between 1.30 and 2.82 in model 1c in Table 5), but an increase in odds of hazardous alcohol use of 30% is still a considerable increase in odds.

The results of the sensitivity analyses performed on the original non-imputed dataset, including complete cases only, showed only some small differences compared to the results of the pooled multiple imputed datasets. The results were largely the same for sexual abuse and violence, bullying and painful or frightening dental treatments, with some additional effects found significant in the complete cases sample. Additionally, the relationship between painful or frightening medical treatments and hazardous alcohol use was significant in childhood and both in childhood and in adulthood in the analyses of the complete cases sample. In the multiple imputed dataset only the latter was significant. However, the results for childhood experiences of painful or frightening medical treatments were almost significant also in the fully adjusted model in the imputed sample, and the beta coefficients were mostly only marginally different in the two samples. As multiple imputation results in more power, corrects for bias under the missing at random (MAR) assumption and even partly corrects for bias under the missing not at random (MNAR) assumption [79], therefore we are confident in the results observed in the multiple imputed dataset.

The practical and clinical implications include the identification of possible PTEs and vulnerable populations that may need screening for hazardous alcohol use in clinical settings, also when dealing with middle-aged and elderly individuals. As an added bonus, this may potentially also lead to a reduction in hazardous alcohol use. Screening for PTEs among individuals with hazardous alcohol use may be just as important, as often individuals with PTE experiences refrain from disclosing this in healthcare settings [10].

### Strengths and limitations

Strengths of this study include the use of a large population-based sample of both women and men, and that the outcome (hazardous alcohol use) is measured with a widely used, validated screening tool for hazardous and harmful alcohol use. In addition, the current study complements previous studies by including a wide range of PTEs, as opposed to specific PTEs such as sexual abuse [66], frightening experiences (natural disasters or terror attacks) [29, 40], and childhood abuse [37]. The broad inclusion of data on PTEs in both childhood and in adulthood gave the opportunity to calculate odds ratios for hazardous alcohol use for each specific PTE, and for investigating whether there was an association between the total sum of PTEs and hazardous alcohol use. Including a sample of adults > 40 years enabled comparisons of PTEs experienced during a lifetime, from childhood to adulthood, as well as studying hazardous alcohol use years after the PTE, despite the cross-sectional design. This also allowed us to differentiate between PTEs experienced in childhood (before age 18) and in adulthood (after age 18), and PTE experienced both in childhood and in adulthood.

However, some limitations need to be accounted for. Population-based studies are prone to selection. Data from non-attenders were not available, but a previous study from the comparable population-based HUNT study in Norway has shown that non-attenders likely have more severe psychological problems and ill health compared to attenders [80]. This may underestimate the effect sizes as those with the heaviest alcohol problems have a higher chance of not attending population-based studies, and potentially introduced a bias of the results [81]. Furthermore, respondents may have been more prone to remember PTEs in adulthood compared to PTEs in childhood due to misclassification. This may have introduced exposure misclassification, as respondents may have reported more PTEs in adulthood. Thus, we cannot exclude that this may have impacted the results in the present study. In the current study, many different associations were examined. To reduce the chance of type 1 error, we decreased the *α*-level to *p* $\le$0.01. Some real associations may thus have been assessed as non-significant due to a conservative alpha level.

## Conclusion

In this cross-sectional population-based sample of middle aged- and elderly, some but not all PTEs were independently associated with an increase in odds of hazardous alcohol use. More specifically, violence and sexual abuse, bullying, painful or frightening medical and dental treatments, increased the odds for hazardous alcohol use. Moreover, as identified in previous studies, several PTE experiences were cumulatively related to hazardous alcohol use. We found an association between the total sum of PTEs and hazardous alcohol use. Future studies may complement this study, by using a longitudinal design, focusing on the role of PTSD following PTEs, and potential interactions between different PTEs in the role of hazardous alcohol use.

## Supplementary Information

Below is the link to the electronic supplementary material.

Supplementary file1 (DOCX 37 KB)

## Data Availability

The datasets generated and/or analysed during the current study are not publicly available as they came from a third party. Legal restrictions protect against potential reverse identification and of de-identified participant information. These data can be made available upon application to the Tromsø Study Data and Publication Committee (The Tromsø Study, Department of Community Medicine, Faculty of Health Sciences, UiT The Arctic University of Norway), e-mail: tromsous@uit.no.

## Abbreviations

ACE: Adverse childhood experiences
AUDIT: Alcohol use disorder identification test
CI: Confidence interval
IPV: Interpersonal violence
OR: Odds ratio
PTE: Potentially traumatic events
PTSD: Post traumatic stress disorder
SUD: Substance use disorder
VIF: Variation inflation factor
