# Supplementary material for: Potentially traumatic events and the association with hazardous alcohol use in 19,128 middle aged and elderly adults: the Tromsø Study 2015–2016
Source: Soc Psychiatry Psychiatr Epidemiol. 2024 Dec 18;60(5):1099–112. doi: 10.1007/s00127-024-02801-3 (PMC12119722; doi:10.1007/s00127-024-02801-3)
Supplement: Supplementary file 1 — Supplementary file1 (DOCX 37 KB) [file 127_2024_2801_MOESM1_ESM.docx]

Supplementary Material, Social Psychiatry and Psychiatric Epidemiology

**Potentially Traumatic Events and the association with Hazardous Alcohol Use: The Tromsø Study 2015-2016**

Vendela Husberg-Bru^1,2, 3*^, Laila A. Hopstock^4^, Jens C. Thimm^1,5^, Torgeir Gilje Lid^2,3^, Kamilla Rognmo^1^, Catharina Elisabeth Arfwedson Wang^1,^ Kristin Gustavson^8,9^

*** Correspondence:** Vendela Husberg-Bru: vendela.husberg-bru@uis.no

| **Table 1:**  Sample characteristics, stratified by sex, based on a multiple imputed sample. The Tromsø Study (2015-2016), N=19 175^a^. | | | | | | | |
| --- | --- | --- | --- | --- | --- | --- | --- |
|  | Women (n=9 903) | | | Men (n=9 272) | | | |
| Mean age | 56.5 (11.0) | | | 57.1 (11.1) | | | |
| Hazardous alcohol use | 5.4 (534) | | | 18.1 (1675) | | | |
| Experienced childhood neglect | 8.0 (793) | | | 5.4 (501) | | | |
| Potentially traumatic events | Childhood  % (n) | Adulthood  % (n) | Experienced both in childhood and in adulthood % (n) | | Childhood   % (n) | Adulthood  % (n) | Experienced both in childhood and in adulthood % (n) |
|  |  |  |  |  | |  |  |
| Serious illness/accident | 4.2 (408) | 16.0 (1557) | .6 (59) | 4.9 (449) | | 20.0 (2144) | 1.1 (99) |
| Sexual abuse | 10.4 (1019) | 4.1 (403) | 1.0 (96) | 3.0 (270) | | 0.4 (42) | 0.1 (4) |
| Violence | 3.9 (383) | 8.8 (862) | 0.5 (53) | 6.1 (561) | | 10.8 (1001) | 1.5 (138) |
| Bullying | 14.0 (1377) | 7.0 (674) | 1.5 (149) | 15.3 (1413) | | 3.8 (350) | 1.5 (137) |
| Witnessed violence | 4.4 (434) | 5.4 (530) | 0.5 (48) | 3.2 (294) | | 4.6 (421) | 0.6 (58) |
| Other frightening, dangerous or violent events | 2.8 (273) | 3.9 (386) | 0.1 (14) | 2.2 (203) | | 6.7 (619) | 0.2 (18) |
| Severe grief after bereavement | 4.1 (407) | 32.1 (3141) | 1.6 (159) | 3.5 (325) | | 23.2 (2134) | 1.1 (103) |
| Painful medical treatment | 3.3 (327) | 8.0 (786) | 0.3 (31) | 3.0 (278) | | 6.6 (611) | 0.2 (19) |
| Painful dental treatment | 18.2 (1778) | 5.3 (519) | 1.1 (104) | 17.6 (1620) | | 5.6 (514) | 1.0 (93) |
| Serious illness/accident of a loved one | 2.7 (246) | 36.7 (3582) | 1.8 (178) | 2.7 (246) | | 28.0 (2568) | 1.6 (151) |
| Numbers are means (Standard deviations) and proportions for continuous and categorical variables.  Sample characteristics, stratified by sex. The Tromsø Study (2015-2016)  ^a^This sample stem from a multiple imputed dataset (n= ranging from 18 915-19 075). Additionally, all participants reporting no alcohol consumption in the previous year, were excluded from the data.  Hazardous alcohol use=AUDIT score >8.  Childhood neglect=Yes/no.  Childhood= Before age 18.  Adulthood= After age 18. | | | | | | | |
|  | | | | | | | |

| **Table 2:**  The association between potentially traumatic events and hazardous alcohol use. N= 17 110. The Tromsø Study (2015-2016). | | | | | | | | | |
| --- | --- | --- | --- | --- | --- | --- | --- | --- | --- |
|  | **Model 1^a^** | | | **Model 1^b^** | | | **Model 1^c^** | | |
|  | **OR** | **99% CI** | ***p*** | **OR** | **99 % CI** | ***p*** | **OR** | **99% CI** | ***p*** |
| **Childhood neglect** | 1.56 | 1.27-1.92 | <.001 | 1.74 | 1.40-2.17 | <.001 | 1.07 | .83-1.36 | .488 |
| **Frightening, dangerous or violent event** Before 18  After 18  Both | .70  1.91  3.38 | .44-1.11  1.53-2.37 1.20-9.51 | .047  <.001 .002 | 1.06 1.57 2.73 | .66-1.70  1.25-1.97  .90-8.22 | .763 <.001 .020 | .74  1.14  1.26 | .45-1.23  .89-1.45  .37-4.24 | .132  .176  .622 |
| **Illness or accidents**  Before 18  After 18  Both | 1.66  1.31  2.93 | 1.29-2.14  1.13-1.52  1.81-4.75 | <.001  <.001 <.001 | 1.48  1.27 2.30 | 1.14-1.92 1.09-1.48 1.39-3.81 | <.001 <.001  <.001 | 1.16  1.00  1.06 | .87-1.53  .85-1.18  .60-1.89 | .174  .988  .774 |
| **Violence**  Before 18  After 18  Both | 2.20  2.86  6.03 | 1.75-2.76  2.44-3.36  4.03-9.00 | <.001  <.001  <.001 | 1.72  2.45  3.74 | 1.36-2.18  2.07-2.90  2.46-5.69 | <.001  <.001  <.001 | 1.35  1.93  2.58 | 1.05-1.74  1.61-2.32  1.61-4.13 | .002  <.001  <.001 |
| **Sexual abuse**  Before 18  After 18  Both | 1.38  1.53  2.43 | 1.12-1.72  1.09-2.16  1.29-4.59 | <.001  <.001  <.001 | 2.28  3.18  5.40 | 1.80-2.88  2.20-4.59  2.79-10.44 | <.001  <.001 <.001 | 1.71  2.00  3.01 | 1.32-2.20  1.34-2.97  1.45-6.26 | <.001  <.001  <.001 |
| **Bullying**  Before 18  After 18  Both | 1.55  1.62  2.34 | 1.33-1.81  1.28-2.06  1.59-3.45 | <.001  <.001  <.001 | 1.34  1.92  2.00 | 1.14-1.58  1.50-2.46  1.34-3.01 | <.001  <.001  <.001 | 1.05  1.40  1.06 | .89-1.26  1.07-1.82  .68-1.67 | .426  <.001  .726 |
| **Witnessed violence**  Before 18  After 18  Both | 1.89  1.61  3.57 | 1.46-2.44  1.26-2.04  2.03-6.27 | <.001  <.001  <.001 | 1.90  1.57  2.78 | 1.45-2.49  1.22-2.01  1.54-5.02 | <.001  <.001  <.001 | 1.25  1.04  1.04 | .92-1.68  .79-1.37  .51-2.11 | .060  .685  .877 |
| **Severe grief after bereavement**  Before 18  After 18  Both | 1.19  1.04  1.98 | .88-1.60  .91-1.19  1.31-2.99 | .129  .482  <.001 | 1.39  1.31  2.34 | 1.02-1.89  1.14-1.51  1.51-3.61 | .006  <.001  <.001 | 1.13  1.12  1.55 | .82-1.57  .96-1.30  .96-2.51 | .320  .062 .020 |
| **Painful medical treatment**  Before 18  After 18  Both | 1.80  1.35  4.05 | 1.36-2.38  1.10-1.67  1.82-9.01 | <.001  <.001  <.001 | 1.93  1.48  4.51 | 1.44-2.60  1.20-1.85  1.92-10.59 | <.001  <.001  <.001 | 1.38  1.12  2.76 | 1.01-1.90  .88-1.42  1.05-7.22 | .009  .223  .007 |
| **Painful dental treatment**  Before 18  After 18  Both | 1.50  1.74  2.17 | 1.30-1.74  1.38-2.19  1.36-3.47 | <.001  <.001  <.001 | 1.57  1.67  2.01 | 1.35-1.82  1.31-2.13  1.23-3.28 | <.001  <.001  <.001 | 1.31  1.37  1.35 | 1.11-1.54  1.06-1.77  .79-2.32 | <.001 <.001 .147 |
| **Illness by close one**  Before 18  After 18  Both | 1.57  1.17  1.81 | 1.14-2.17  1.03-1.33  1.23-2.66 | <.001  .002  <.001 | 1.54  1.32  1.68 | 1.10-2.15  1.16-1.51  1.12-2.52 | <.001  <.001  <.001 | 1.23  1.11 .92 | .87-1.76  .96-1.28  .58-1.45 | .120  .065  .648 |
| OR=Odds ratio, CI= Confidence interval.  The association between potentially traumatic events and hazardous alcohol use The Tromsø Study (2015-2016).  The estimates are results from the original dataset (n=17 110).  Hazardous alcohol use= Alcohol Use Disorder Identification Test (AUDIT) >8.  The sample is from a original non-imputed dataset, participants who abstained from alcohol in the past 12 months were excluded from the analyses.  * α level= .01.  a Adjusted for age and sex.  b Adjusted for age and sex, and all PTEs included in the analysis. | | | | | | | | | |
